# Novel Gene Rearrangement in the Mitochondrial Genome of Three *Garra* and Insights Into the Phylogenetic Relationships of *Labeoninae*

**DOI:** 10.3389/fgene.2022.922634

**Published:** 2022-06-08

**Authors:** Chi Zhang, Kun Zhang, Ying Peng, Jianshe Zhou, Yifan Liu, Bingjian Liu

**Affiliations:** ^1^ Institute of Fisheries Science, Tibet Academy of Agricultural and Animal Husbandry Sciences, Lhasa, China; ^2^ National Engineering Laboratory of Marine Germplasm Resources Exploration and Utilization, Zhejiang Ocean University, Zhoushan, China

**Keywords:** *Garra*, gene rearrangement, complete mtDNA sequence, *Labeoninae*, phylogeny

## Abstract

Complete mitochondrial genomes (mitogenomes) can provide valuable information for phylogenetic relationships, gene rearrangement, and molecular evolution. Here, we report the mitochondrial whole genomes of three *Garra* species and explore the mechanisms of rearrangements that occur in their mitochondrial genomes. The lengths of the mitogenomes’ sequences of *Garra dengba*, *Garra tibetana*, and *Garra yajiangensis* were 16,876, 16,861, and 16,835, respectively. They contained 13 protein-coding genes, two ribosomal RNAs, 22 transfer RNA genes, and two identical control regions (CRs). The mitochondrial genomes of three *Garra* species were rearranged compared to other fish mitochondrial genomes. The *tRNA-Thr*, *tRNA-Pro* and CR (*T-P-CR*) genes undergo replication followed by random loss of the *tRNA-Thr* and *tRNA-Pro* genes to form tRNA-Thr, CR1, tRNA-Pro and CR2 (*T-CR-P-CR*). Tandem duplication and random loss best explain this mitochondrial gene rearrangement. These results provide a foundation for future characterization of the mitochondrial gene arrangement of *Labeoninae* and further phylogenetic studies.

## Introduction

The subfamily Labeoninae is wildly distributed in Asia and Africa as one of the most diverse freshwater fishes in the family Cyprinidae (Cypriniformes). About 26 genera are distributed in China, 12 of which are endemic, with a great diversity of genera and species (Pei-Qi and Een-Duan, 1984). Once, Howes et al. (1991) and Winfield et al. (2012) proposed that the tribe Labeonini is essentially equivalent to the subfamily Labeoninae in Cyprininae (Howes et al., 1991; Winfield and Nelson, 2012). Because of the great diversity of Labeoninae orofacial morphology, it is often used as a key feature for identifying the included genera and for recovering the phylogenetic history hypothesis suite. However, since the morphological diversity and the difficulty of distinguishing the ambiguous morphological characteristics, the taxonomy of the Labeoninae has been constantly subject to revision, and the phylogenetic relationships within them remain amid change. Although some researchers erected a few new genera (e.g., *Hongshuia*, *Qianlabeo*, and *Stenorhinchus*) (Zhang and Chen, 2004; Zhang and Fang, 2005; LI et al., 2006; Zhang et al., 2008), and rearranged the generic assignment of some species based on the oromandibular morphology (Yuan et al., 2008). There was no input from molecule-based phylogenetic evidence. Furthermore, Yang and Mayden (2010) constructed the most comprehensive molecular phylogeny just including one species of each of 11 genera, supporting the monophyly of Labeoninae and proposing a model for the relationships of the major sublineages within Labeoninae (Yang and Mayden, 2010). However, limited by the number of mitogenomes, the monophyly of many genera and the validity of the nomenclature are unresolved. Therefore, re-establishing the Labeoninae phylogenetic relationship using suitable species is necessary.

*Garra* is a genus of fish in the carp family of the subfamily Labeoninae, distributed in southern China, across Southeast Asia, India, and the Middle East to northern and central Africa (STIASSNY and GETAHUN, 2007). *Garra* is usually found in swift-flowing streams, where they hold on to rocks with their highly modified mouths like suckers, including bottom-dwelling fishes. They feed on plant debris in open-water habitats and on periphyton in bottom-surface habitats (Kullander and Fang, 2004; Nebeshwar et al., 2009). Currently, 20 different *Garra* species have been identified from the Brahmaputra River basin, three of which in China, namely, *G. dengba*, *G. Tibetan*, and *G. yajiangensis* caught our attention due to the rearrangement in their mitochondrial genome (Deng et al., 2018; Gong, 2018; Gong et al., 2018; Zhang et al., 2020). They were all collected from high altitudes. Among them, *G. dengba* is derived from the Chayu-Qu, a stream that flows into the Brahmaputra River in eastern Tibet, China. It shares with the five *Garra* fishes (*G. arupi*, *G. elongata*, *G. gravelyi*, *G. kalpangi*, and *G. rotundinasus*) an incipient proboscis on the nose, but differs from them in its less divergent dorsal and anal fins and more perforated lateral line scales (Deng et al., 2018). Meanwhile, due to the similarity of morphological characters, *G. tibetana* was for a long time mistakenly identified as *G. kempi* (Galtier et al., 2009). *G. yajiangensis*, a member of the long-nosed species group, is distinguished from other members of the group mainly by the presence of a prominent, quadrangular, slightly bilobed proboscis. Apart from *G. tibetana* has been reported as a complete mitochondrial sequence, current researches on these three *Garra* fishes are mainly stagnant in morphology while their corresponding molecular evolutions are still limited (Zhang et al., 2020). In addition, Zhang et al. (2020) identified two control regions in the mitochondrial genome of *G. tibetana* (Zhang et al., 2020). Also, Li et al. (2016) reported that the *tRNA-Pro* gene of *G. kempi* is positioned between two control regions, and a 246 bp repeat unit is identified in the second control region, which is different from the typical mitochondrial genome organization in vertebrate (Li et al., 2016). However, He et al. (2016) reported that the complete mitochondrial genome of *G. imberba* is a classic structure (He et al., 2016). A different mitochondrial genome composition is present in *Garra* fish, and no one has yet identified the reason for this phenomenon. Therefore, this study reports novel gene rearrangement in the mitochondrial genome of *G. dengba*, *G. tibetana*, and *G. yajiangensis*, which could provide important genomic information for studying the evolutionary relationships and population genetics of the genus *Garra*.

The mitochondrial genome (Mitogenome) houses its DNA and encodes many key proteins for the assembling and activation of the mitochondrial respiratory complex (Yan et al., 2019). Mitogenomes as a molecule marker were used for species classification, population genetics, molecular systematic geography, molecular ecology, and other areas (Groves and Shields, 1996; Nielsen et al., 2010; Cheng et al., 2012; Zhang et al., 2018). Vertebrate mitogenomes are generally 16–20 kb in length (Boore, 1999), compared to plant mitochondrial genomes, which are shorter. The structure of the vertebrate mitogenome typically consists of 13 protein-coding genes (PCGs), 22 tRNA genes (tRNAs), and two rRNA genes (rRNAs) (Boore, 1999). In the vertebrate mitogenome, the order of these genes is conserved and generally unaltered (Bibb et al., 1981; Roe et al., 1985). The mitochondrial genome is inherited maternally in most animals and therefore has a very low rate of recombination (Brown et al., 1979). However, variants of the gene sequence have now been identified in a variety of vertebrates, including amphibians, reptiles, birds, marsupials, and fish (Pääbo et al., 1991; Macey et al., 1997; Eberhard and Wright, 2015; Liu et al., 2019a; Lü et al., 2019). In recent years, with the advancement of sequencing techniques, more and more mitochondrial rearrangements are being identified.

In general, the structure of the fish mitochondrial genome is extremely conserved (Gong et al., 2013). With the gradual increase in mitochondrial DNA sequence data for fish, reports of mitogenome rearrangements continue to appear (Miya et al., 2001; Inoue et al., 2003; Zhang et al., 2021a). To date, a few models have been employed for mitochondrial gene rearrangement. These models include the tandem duplication/random loss (TDRL) (Moritz and Brown, 1987), recombination model (Lunt and Hyman, 1997), tRNA mismatch model (Cantatore et al., 1987), tandem duplication/nonrandom loss model (RDNL) (Lavrov et al., 2000) and double replication/random loss model (DRRL) (Shi et al., 2014). The TDRL model occurs by tandem duplication of certain genes that undergo rearrangement, followed by random deletion of repetitive sequences. The model has been widely applied to explain the translocation of genes encoded on the same strand (Shi et al., 2015; Wang et al., 2018). The RDNL model differs from TDRL in non-random loss and is dependent on the transcriptional polarity and location of the gene.

In this study, three mitogenomes of *Garra* species (*G. dengba*, *G. tibetana*, and *G. yajiangensis*) were sequenced and annotated. We have also characterized their mitochondrial genome structure and phylogenetic analysis within their subfamily Labeoninae. In addition to this, we have explored the mechanisms by which their mitochondrial genes undergo rearrangement. Based on the above analysis, we hope that these results will provide a better understanding of the mechanisms by which rearrangements occur in the mitochondrial genomes of *Garra* species, as well as their evolution.

## Materials and Methods

### Sample Collection, DNA Extraction, and Sequencing

The sample of *G. dengba* was collected in Chayu City, Tibet, China (28°66′ N, 97°46′ E). Samples of *G. tibetana* and *G. yajiangensis* were collected in Motuo City, Tibet, China (29°32′ N, 95°33′ E). Total genomic DNA was extracted from muscle tissue using the Qiagen QIAamp tissue kit according to the manufacturer’s protocol. The DNA sediment was solubilized in double-distilled water, stored at 4°C, and then quantified in concentration. The *G. tibetana* mitogenome was amplified with ten pairs of universal primer by general PCR. The general PCR cycle requirement for DNA amplification is 5 min at 94°C, [30 s at 94°C, 30 s at 55–56°C, 1 min at 72°C] X 35 cycles, and 10 min at 72°C. Sequences were sequenced using an ABI Genetic Analyzer (Applied Biosystems, China). Complete mitogenome sequencing of *G. dengba* and *G. yajiangensis* was performed on an Illumina HiSeq X Ten platform.

### Assembly, Annotation, and Analysis

The acquired sequence PCR fragments were processed through CodonCode Aligner 9.0.1 (CodonCode Corporation, Dedham, MA) and the complete mitochondrial genome was assembled. Clean data without sequencing adapters were assembled *de novo* using NOVOPlasty software (Dierckxsens et al., 2016). The new mitogenome was annotated with the vertebrate mitochondrial code using the MITOS (Donath et al., 2019). The structures of the 22 tRNAs were determined using tRNA-scan 2.0 (Chan et al., 2021) and then the tRNA structures were mapped by online web software Forna (Kerpedjiev et al., 2015). The circular map of the mitochondrial genome was produced by using the OGDRAW v1.3.1 (Greiner et al., 2019). Base composition and relative synonymous codon usage (RSCU) for 13 PCGs of *Garra* were computed and sorted using MEGA X (Kumar et al., 2018). Composition skew values were calculated by the following formulas: AT skew = (A—T)/(A + T); GC skew = (G—C)/(G + C) (Perna and Kocher, 1995). The putative origin of L-strand replication (OL) was identified by the Mfold Web Server (Zuker, 2003).

### Phylogenetic Analyses

Rapid identification of 12 PCGs in the mitochondrial genome was performed using DAMBE v7.2.3 software (Xia, 2018). 13 PCGs were used excluding ND6 owing to its heterogeneous base composition and poor consistent phylogenetic performance (Miya et al., 2003). Sequences were compared using the default parameters of Clustal X 2.0 (Larkin et al., 2007). The ambiguous sequences were eliminated by Gblock (Talavera and Castresana, 2007). Subsequently, the results of the multiple sequence comparisons were then used to construct phylogenetic trees based on maximum likelihood (ML) and Bayesian inference (BI) analyses. The ML tree was built in PhyML 3.0 (Guindon et al., 2010), using the best model (TVM + F + R8) selected in ModelFinder with 1000 nonparametric bootstrap replications (Kalyaanamoorthy et al., 2017). Bayesian inference (BI) methods were used with the program MrBayes 3.2 (Ronquist et al., 2012). Based on the Akaike information criteria (AIC), MrModeltest 2.2 (Nylander et al., 2004) was performed to determine the best evolutionary model among 24 models for BI analysis and pointed out that GTR + G + I was the analytical data set for the best-fit substitution model. BI analysis was performed using Markov chain Monte Carlo (MCMC), sampled every 1,000 generations each with three heated chains and one cold chain run for 6,000,000 generations, and the first 25% of the burns were discarded. Visualization of the tree was realized using online web iTOL v5 (https://itol.embl.de) (Letunic and Bork, 2021).

## Results and Discussion

### Genome Organization and Composition

The complete mitochondrial genomes of three *Garra* (*G. dengba*, accession no. OL826794; *G. tibetana* accession no. NC_045032 and *G. yajiangensis*, accession no. OL826795) in GenBank were 16,876, 16,861 and 16,835 bp in length, respectively (Table 1). The structure of the mitochondrial genomes of these three fish species is extremely similar. The mitochondrial genomes of all three species are very similar in structure, containing 13 protein-coding genes (PCGs), two rRNAs, 22 tRNAs, a light-stranded replication initiation region (OL), and two control regions (CR). Compared to the structure of the mitochondrial genomes of most teleost fishes, the mitochondrial genomes of the three fishes in this study have an extra CR. The main reason for this is the rearrangement of mitochondrial genes in these three fish species (Figure 5). The presence of replicating CR was considered a special feature in the vertebrate mitochondrial genome (Jiang et al., 2007; Shi et al., 2013). The nucleotide composition of *G dengba*, *G tibetana,* and *G yajiangensis* mitogenomes had a higher A + T bias of 57.42, 58.44, and 58.44%, respectively, and both showed positive AT-skew and negative GC-skew (Table 2).

**TABLE 1:** Summary of gene/element feature of *Garra dengba* (GD), *Garra tibetana* (GT) and *Garra yajiangensis* (GY).

Gene	Poistion start	Poistion end	Size (bp)	Intervening spacer (bp)*	Start codon	Stop codon	Strand
	GT/GD/GY						
tRNA-Phe	1/1/1	69/68/69	69/68/69	0/0/0			H
12S rRNA	70/69/70	1021/1019/1019	952/951/950	0/0/0			H
tRNA-Val	1021/1020/1020	1092/1091/1091	72/72/72	0/0/0			H
16S rRNA	1093/1092/1092	2780/2779/2778	1688/1688/1687	0/0/0			H
tRNA-Leu	2781/2780/2779	2856/2855/2854	76/76/76	0/1/1			H
ND1	2857/2857/2856	3831/3831/3830	975/975/975	3/4/4	ATG/ATG/ATG	TAA/TAA/TAA	H
tRNA-Ile	3835/3836/3835	3906/3907/3906	72/72/72	−2/−2/−2			H
tRNA-Gln	3905/3906/3905	3975/3976/3975	71/71/71	1/1/1			L
tRNA-Met	3977/3978/3977	4045/4046/4045	69/69/69	0/0/0			H
ND2	4046/4047/4046	5092/5091/5092	1047/1045/1047	−2/0/−2	ATG/ATG/ATG	TAG/T--/TAG	H
tRNA-Trp	5091/5092/5091	5161/5162/5161	71/71/71	2/2/2			H
tRNA-Ala	5164/5165/5164	5232/5233/5231	69/69/68	1/1/1			L
tRNA-Asn	5234/5235/5233	5306/5307/5305	73/73/73	0/0/0			L
OL	5307/5306/5306	5340/5341/5338	34/34/33	0/0/0			H
tRNA-Cys	5341/5342/5339	5406/5409/5405	66/68/67	1/1/1			L
tRNA-Tyr	5408/5411/5407	5478/5481/5477	71/71/71	1/1/1			L
COI	5480/5483/5479	7030/7033/7029	1551/1551/1551	0/1/0	GTG/GTG/GTG	TAA/TAA/TAA	H
tRNA-Ser	7031/7035/7030	7101/7103/7100	71/69/71	3/4/3			L
tRNA-Asp	7105/7108/7104	7174/7177/7174	70/70/71	13/12/12			H
COII	7188/7190/7187	7878/7880/7877	691/691/691	0/0/0	ATG/ATG/ATG	T--/T--/T--	H
tRNA-Lys	7879/7881/7878	7954/7956/7953	76/76/76	1/1/1			H
ATP8	7956/7958/7955	8120/8122/8119	165/165/165	−7/−7/−7	ATG/ATG/ATG	TAG/TAA/TAG	H
ATP6	8114/8116/8113	8797/8799/8796	684/684/684	−1/−1/−1	ATG/ATG/ATG	TAA/TAA/TAA	H
COIII	8797/8799/8796	9582/9583/9581	786/785/786	−1/0/−1	ATG/ATG/ATG	TAA/TA-/TAA	H
tRNA-Gly	9582/9584/9581	9653/9654/9652	72/71/72	0/0/0			H
ND3	9654/9655/9653	10004/10003/10001	351/349/349	−2/0/0	ATG/ATG/ATG	TAG/T--/T--	H
tRNA-Arg	10003/10004/10002	10072/10073/10071	70/70/70	0/0/0			H
ND4L	10073/10074/10072	10369/10370/10368	297/297/297	−7/−7/−7	ATG/ATG/ATG	TAA/TAA/TAA	H
ND4	10363/10364/10362	11743/11744/11742	1381/1381/1381	−2/0/0	ATG/ATG/ATG	T--/T--/T--	H
tRNA-His	11744/11745/11743	11812/11814/11811	69/70/69	0/0/0			H
tRNA-Ser	11813/11815/11812	11881/11883/11879	69/69/68	1/1/1			H
tRNA-Leu	11883/11885/11881	11955/11957/11953	73/73/73	3/3/3			H
ND5	11959/11961/11957	13782/13784/13780	1824/1824/1824	−3/−4/−4	ATA/ATA/ATA	TAA/TAA/TAA	H
ND6	13780/13781/13777	14300/14302/14298	521/522/522	0/0/0	ATG/ATG/ATG	TA-/TAA/TAG	L
tRNA-Glu	14301/14303/14299	14369/14371/14367	69/69/69	4/5/5			L
Cytb	14374/14377/14373	15514/15517/15509	1141/1141/1137	0/0/4	ATG/ATG/ATG	T--/T--/TAA	H
tRNA-Thr	15515/15518/15514	15586/15589/15585	72/72/72	0/0/0			H
D-Loop1	15587/15590/15586	16524/16490/16499	938/901/914	0/0/0			H
tRNA-Pro	16525/16491/16500	16594/16560/16568	70/70/79	0/0/0			L
D-Loop2	16595/16561/16569	16876/16861/16835	282/301/267	0/0/0			H

**TABLE 2:** Composition and skewness of *Garra dengba* mitogenome.

Gene	A	T	C	G	A + T (%)	G + C (%)	AT-skew	GC-skew	Length (bp)
Mitogenome	29.36	28.07	26.86	15.72	57.42	42.58	0.0224	−0.2616	11,412
ND1	29.85	25.74	29.64	14.77	55.59	44.41	0.0738	−0.3349	975
ND2	32.22	23.14	31.26	13.38	55.35	44.65	0.1641	−0.4004	1,046
COI	26.95	30.56	24.63	17.86	57.51	42.49	−0.0628	−0.1593	1,551
COII	31.4	26.92	26.05	15.63	58.32	41.68	0.0769	−0.25	691
ATP8	34.55	27.88	26.06	11.52	62.42	37.58	0.1068	−0.3871	165
ATP6	29.09	30.41	26.17	14.33	59.5	40.5	−0.0221	−0.2924	684
COIII	27.61	26.34	28.75	17.3	53.94	46.06	0.0236	−0.2486	786
ND3	27.92	28.49	27.92	15.67	56.41	43.59	−0.0101	−0.281	351
ND4L	24.92	25.93	31.31	17.85	50.84	49.16	−0.0199	−0.274	297
ND4	31.43	27.23	27.52	13.83	58.65	41.35	0.0716	−0.331	1,381
ND5	32.8	26.93	27.32	12.95	59.74	40.26	0.0983	−0.3569	1,823
ND6	13.24	40.88	12.67	33.21	54.13	45.87	−0.5106	0.4477	521
Cytb	29.89	29.1	26.64	14.37	58.98	41.02	0.0134	−0.2991	1,141
rRNAs	34.98	20.27	24.25	20.5	55.25	44.75	0.2661	−0.0838	2,639
tRNAs	28.78	27.05	20.77	23.4	55.83	44.17	0.031	0.0595	1,560
CR1	35.07	31.02	20.36	13.54	66.10	33.9	0.0613	−0.2013	938
CR2	45.04	28.72	21.63	4.61	73.76	26.24	0.2212	−0.6486	282

### Protein-Coding Genes and Codon Usage

Like the typical mitochondrial genome of vertebrates (Zhang et al., 2021b; Liu et al., 2019b), the 13 PCGs consist of one cytochrome b (*Cyt b*), two ATPases (*ATP6* and *ATP8*), and three cytochrome c oxidases (*COI-COIII*), and seven NADH dehydrogenases (*ND1-ND6* and *ND4L*). Twelve PCGs (*ND1*, *ND2*, *COI*, *COII*, *ATP8*, *ATP6*, *COIII*, *ND3*, *ND4L*, *ND4*, *ND5*, and *Cytb*) were coded on the heavy strand (H-strand) and the remaining *ND6* gene was coded on the light strand (L-strand) (Table 1). All PCGs use the start codon ATG except for COI, which uses GTG. Most of the 13 PCGs ended with TAA or TAG, whereas four other PCGs (*COI*, *ND4*, *ND6,* and *Cytb*) use a single T-- or TA- as the stop codon (Table 1). The incomplete stop codon has also been found in all other *Garra* species (Su et al., 2015; Xiong et al., 2016). The presence of incomplete stop codons is a common phenomenon in invertebrate and vertebrate mitochondrial genes (Lin et al., 2020; Zhang et al., 2021c). All 12 PCGs genes (*ND1-ND3*, *COI-COIII*, *ATP8*, *ATP6*, *ND4L*, *ND4*, *ND5*) in the three *Garra* mitochondria had negative GC-skew values and they were all encoded on the H-strand, in addition, the *ND6* gene encoded on the L-strand had a positive GC-skew value (Table 2; Supplementary Table S1, S2).

**FIGURE 1:**
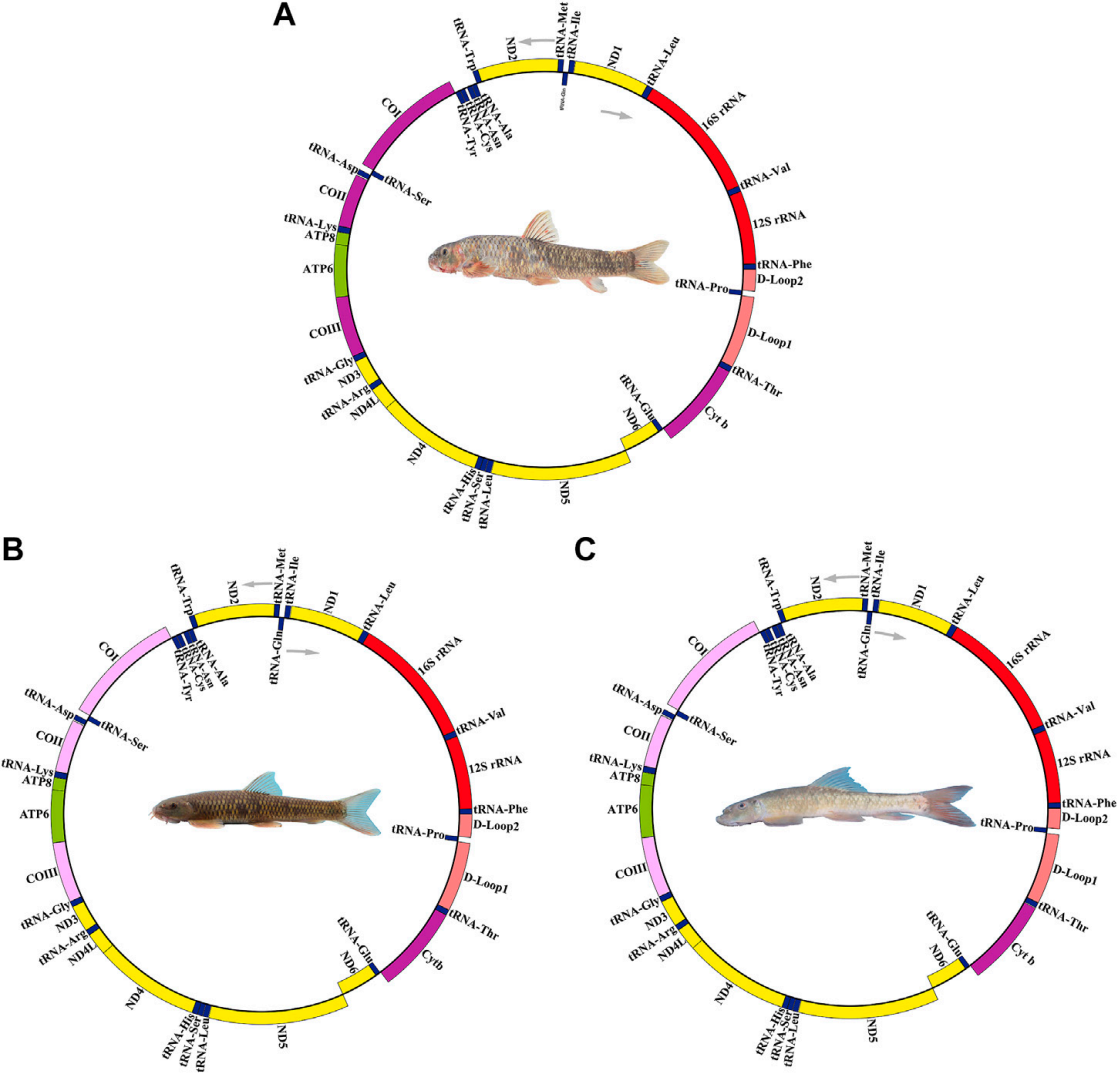
The RSCU of the mitogenome in **(A)** *Garra dengba*, **(B)** *Garra tibetana* and **(C)** *Garra yajiangensis*. The RSCU of the three Garra species mitogenomes show that the usage of NNA and NNC (N for A, T, C, G) was more frequent than NNT and NNG in the G. dengba, G. tibetana, and G. yajiangensis.

Depending on the codon degeneration pattern, the amino acids serine and leucine are encoded by six synonymous codons, and the remaining amino acids are encoded by four or two codons. A count of the translated amino acid content of 13 PCGs was found. The top utilized amino acids are Leu, Ile, and Ala, and the few top utilized amino acids are Asp, Arg, and Cys (Figure S1). This phenomenon is also commonly seen in the mitochondrial genomes of other fish (Su et al., 2015; Zhang et al., 2021b). We measured the RSCU of the three *Garra* species mitogenomes (Figure 1) and the results showed that the usage of NNA and NNC (N for A, T, C, G) was more frequent than NNT and NNG in the *G. dengba*, *G. tibetana,* and *G. yajiangensis*.

### Transfer and Ribosomal RNAs

**FIGURE 2:**
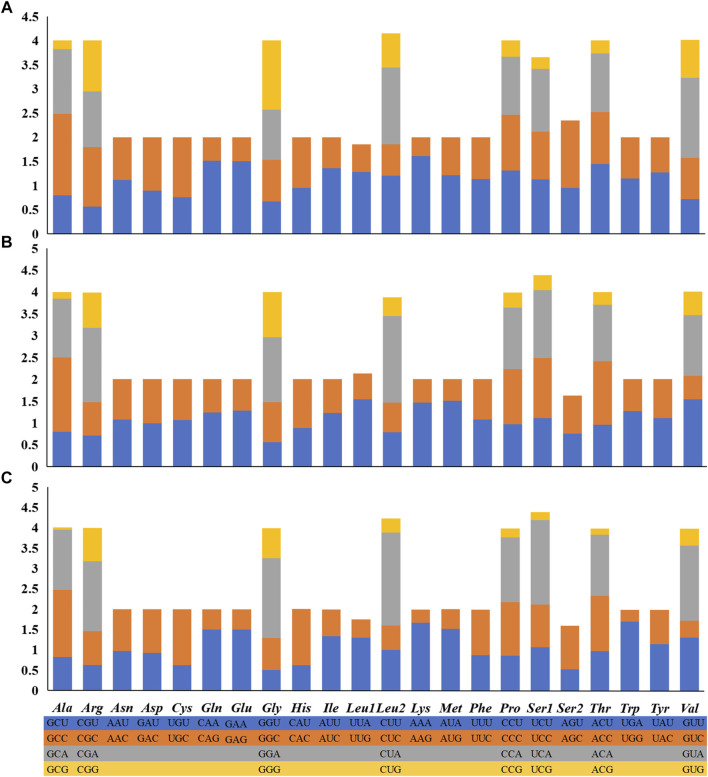
Mitochondrial genome maps of the three *Garra* in this study. **(A)** *Garra dengba* mitochondrial genome visualization ring diagram. **(B)** *Garra tibetana* mitochondrial genome visualization ring diagram. **(C)** *Garra yajiangensis* mitochondrial genome visualization ring diagram. Genes encoded on the heavy or light strands are shown at the outside or inside of the circular gene map, respectively. The mitochondrial genome visualization ring diagram of the three species show the same structure and all have two control regions.

Like the typical set of tRNA genes in fish mitogenomes, all three *Garra* species mitogenomes included 22 tRNAs, a 12S rRNA, and 16S rRNA. Of these 22 tRNAs, 14 tRNAs were encoded on the H-strand and the remaining eight (*tRNA-Gln*, *tRNA-Ala*, *tRNA-Asn*, *tRNA-Cys*, *tRNA-Tyr*, *tRNA-Ser*, *tRNA-Glu,* and *tRNA-Pro*) were encoded on the L-strand (Figure 2). All of them had two kinds of *tRNA-Leu* and *tRNA-Ser* (Table 1). Secondary clover structure of tRNA genes identified in the mitogenome of *G. dengba*, *G. tibetana*, and *G. yajiangensis* were shown in Supplementary Figures S2–4. Out of 22 tRNAs, all have a typical cloverleaf structure except for *tRNA-Ser* (GCT), which lacks the entire dihydrouridine arm. The 12S rRNA gene was positioned between *tRNA-Phe* and *tRNA-Val*, and the 16S rRNA gene was located between *tRNA-Val* and *tRNA-Leu* (UUR). The lengths of 12S rRNA were 952, 951, and 950 in the mitochondrial genomes of *G. dengba*, *G. tibetana,* and *G. yajiangensis*, respectively. The size of the 16S rRNA in *G. dengba* and *G. tibetana* were 1,688 bp, both a little longer than in *G. yajiangensis* (1,687bp). Positive AT-skew values for both tRNA and rRNA in the mitochondrial genomes of *G. dengba*, *G. tibetana,* and *G. yajiangensis* indicate that the As content is higher than the Ts content (Table 2, Supplementary Tables S1, S2).

### L-Strand Origin of Replication and Control Region

**FIGURE 3:**
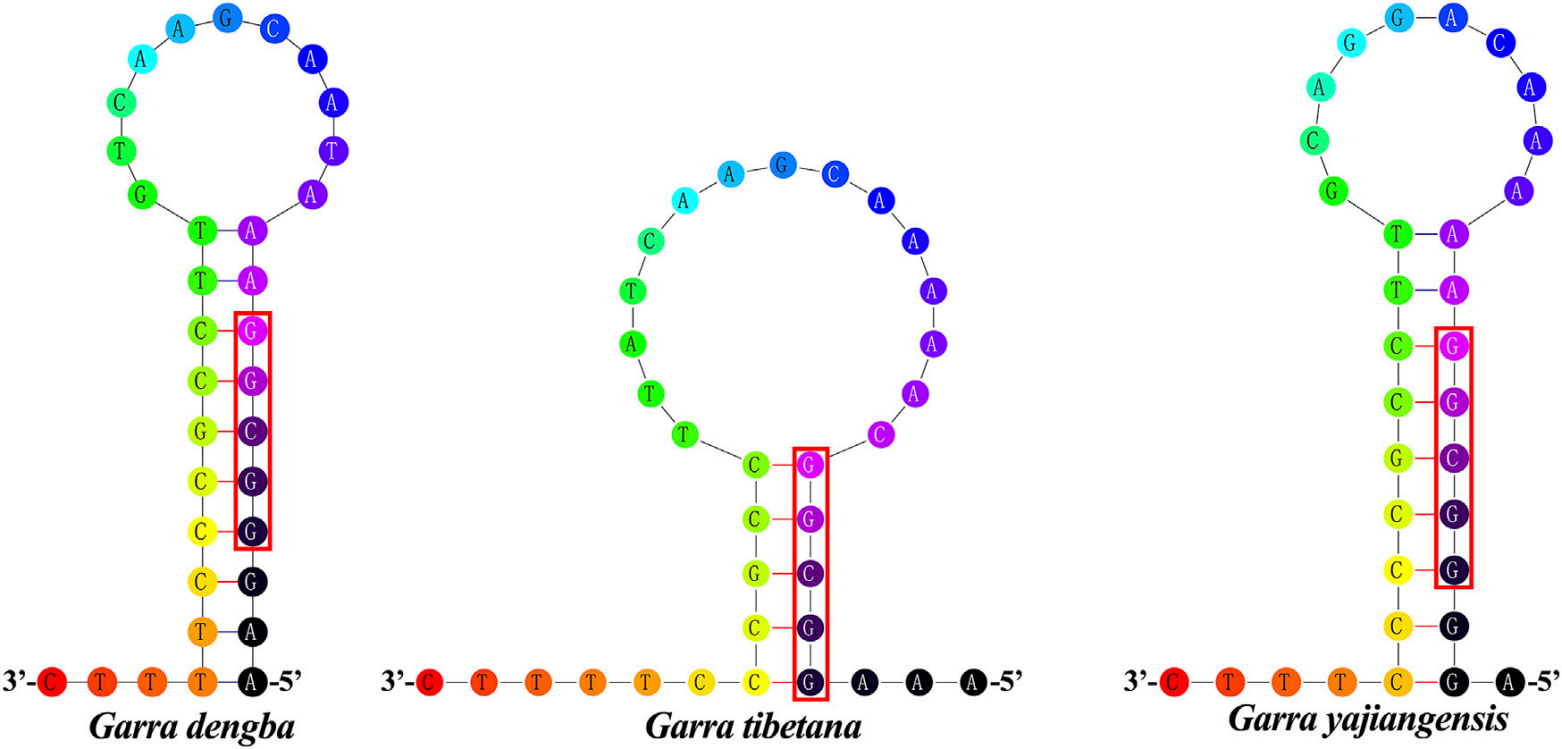
Inferred secondary structures of the OL of *Garra* mitogenome, **(A)** *Garra dengba*, **(B)** *Garra tibetana*, and **(C)** *Garra yajiangensis*. The OLs of the three *Garra* mitogenomes were located between *tRNA-Asn* and *tRNA-Cys*, and *G. dengba* and *G. tibetana* were of the same length (34 bp) and longer than *G. yajiangensis* (33 bp).

The origin of light chain replication (OL) was usually found within a WANCY group and can fold into a stem-loop ring secondary structure (Zhang et al., 2021a). The OL of *G. dengba* and *G. tibetana* were located between *tRNA-Asn* and *tRNA-Cys* with the same length of 34 bp, and 1 bp longer than *G. yajiangensis* (33 bp) (Table 1). The two-structure prediction of the OL region revealed that the stem lengths in the OL regions of *G. dengba*, *G. tibetana,* and *G. yajiangensis* were 10, 5, and 9 bp, respectively, and the loops were 11, 15, and 10nt, respectively (Figure 3). In these OL structures, the utilization of stem codons showed a clear asymmetry, with more pyrimidines at the 5′ end of the sequence. In contrast to other fish studies (Catanese et al., 2010), the conserved sequence 5′-GCCGG-3′ was not present in the OL region in this study, but rather a segment of the 5′-GGCGG-3′ sequence was present.

**FIGURE 4:**
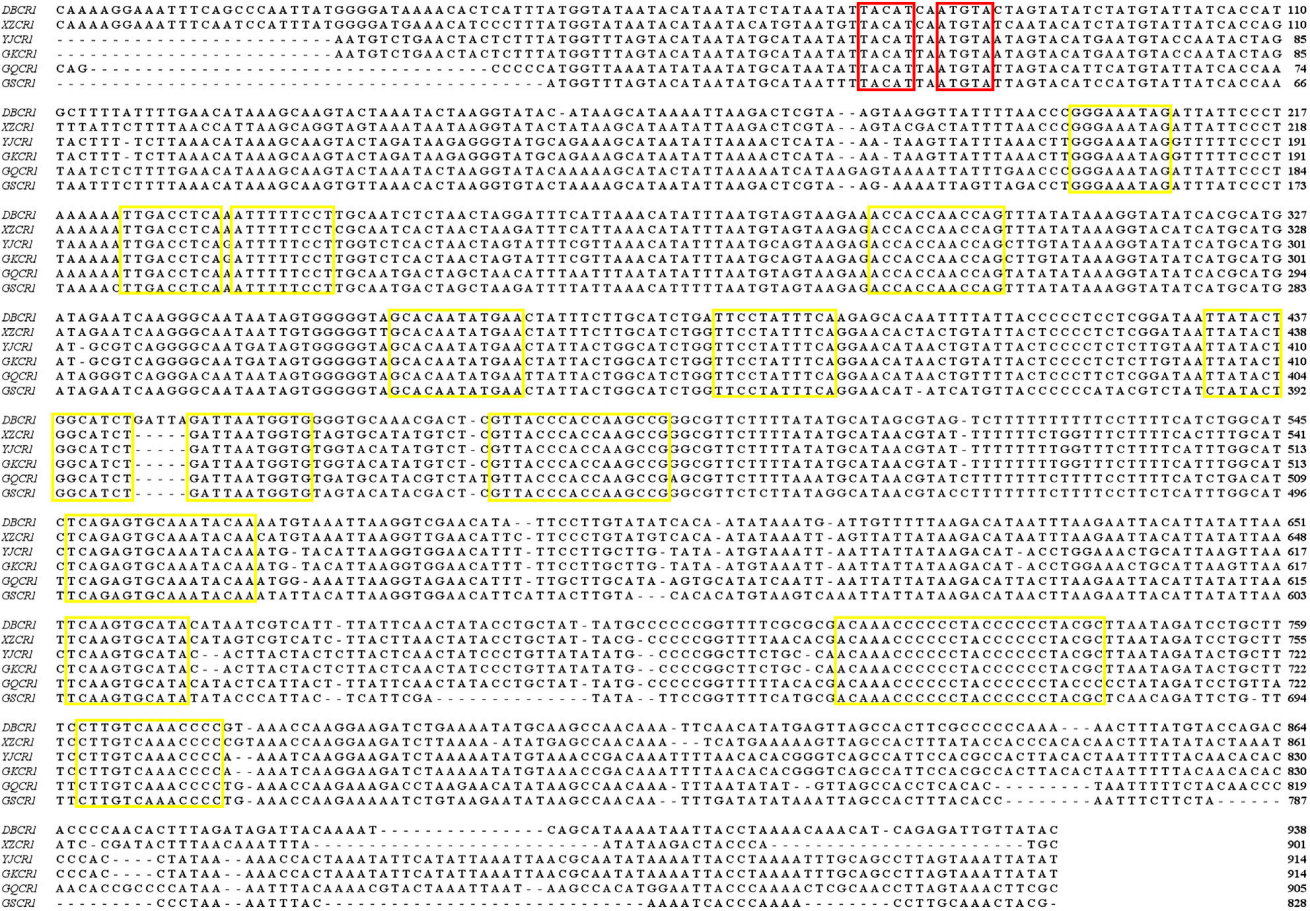
Compositional features of the first control region of the rearranged *Garra* mitochondrial genome. Palindromic motif sequences “TACAT” and “ATGTA” are marked in red boxes. Conservative sequences are marked using yellow boxes. *DBCR1*: The first control region of *Garra dengba. XZCR1*: The first control region of *Garra tibetana. YJCR1*: The first control region of *Garra yajiangensis. GKCR1*: The first control region of *Garra kempi*. *GQCR1*: The first control region of *Garra qiaojiensis. GSCR1*: The first control region of *Garra salweenica*.

The *CR* is the greatest non-coding region in the fish mitogenome which is analogous to the A + T enriched region of the insect mitogenome (Liu et al., 2013). The CR1 and CR2 of *Garra* mitogenomes were located between *tRNA-Thr* and *tRNA-Pro*, *tRNA-Pro*, and tRNA-*Phe*, respectively (Figure 1). The lengths of CR1 in the three *Garra* mitogenomes were 938, 901, and 914 bp (Table 1), and the ratio of AT was 66.10, 66.37, and 65.75%, respectively (Table 2, Supplementary Tables S1, S2). The length of CR2 in the three *Garra* mitogenomes was 282, 301, and 267 bp (Table 1), and the ratio of AT was 73.76, 71.43, and 73.41%, respectively (Table 2, Supplementary Tables S1, S2). The palindromic sequence motifs “TACAT” and “ATGTA” related to heavy chain replication termination were found in both CRs. The “TACAT” and “ATGTA” motifs are generally referred to as the terminator region because of their ability to form a stable hairpin structure (Figure 4, in the red box). Six *Garra* CRs were compared to probe the conservation of sequences. The results revealed 12 conserved blocks in Figure 4 (In the yellow box). As far as we know, this is the first report of a study reporting conserved blocks of the CR in *Garra* mitogenomes.

### Gene Rearrangement

**FIGURE 5:**
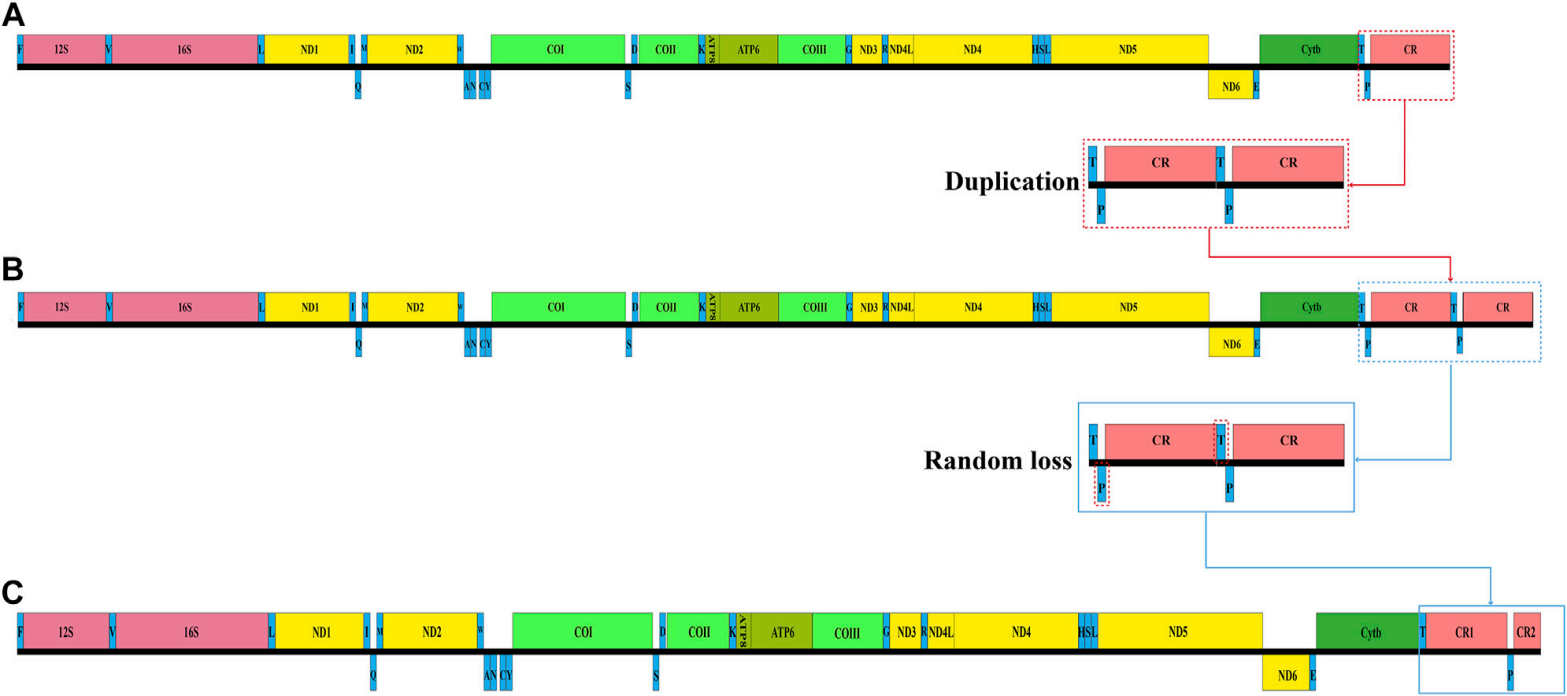
Analysis of *Garra* mitochondrial gene rearrangement. **(A)** The typical mitochondrial genome organization in *Garra*. **(B)** The normal mitochondrial gene blocks (*T-P-CR*) form gene blocks (*T-P-CR-T-P-CR*) after complete duplication. **(C)** The rearrangement mitochondrial genome organization of *Garra* species. The replicated CR is left behind in some genes (T and P) that undergo random loss events. *F*: *tRNA-Phe*; *V*: *tRNA-Val*; *L*: *tRNA-Leu*; *I*: *tRNA-Ile*; *Q*: *tRNA-Gln*; *M*: *tRNA-Met*; *W*: *tRNA-Trp*; *A*: *tRNA-Ala*; *N*: *tRNA-Asn*; *C*: *tRNA-Cys*; *Y*: *tRNA-Tyr*; *S*: *tRNA-Ser*; *D*: *tRNA-Asp*; *K*: *tRNA-Lys*; *G*: *tRNA-Gly*; *R*: *tRNA-Arg*; *H*: *tRNA-His*; *S*: *tRNA-Ser*; *L*: *tRNA-Leu*; *E*: *tRNA-Glu*; *T*: *tRNA-Thr*; *P*: *tRNA-Pro*.

By comparing the structure of the mitochondrial genomes of other vertebrates (Kim et al., 2004), we found that the mitochondrial genomes of the three *Garra* species had undergone partial recombination of genes. The two genes that changed position in the mitochondrial genome of these three *Garra* species were *tRNA-Pro* and *CR*. In general, the *tRNA-Pro* was positioned between the *tRNA-Thr* and CR, and only one CR region was positioned at the end of the mitogenome. However, the position of *tRNA-Pro* in the mitochondrial genome of these three *Garra* species changed, and a CR was copied (Figure 5). In this study, the gene sequences were identical to those of the vertebrate mitochondrial genome, except for the *tRNA-Pro* translocation and CR repeats.

To better characterize the mechanics of gene rearrangement that occur in the *Garra* mitochondrial genome, we have explored this in-depth. Several models have been proposed for the mechanism of rearrangement of the mitochondrial genome (Poulton et al., 1993; Arndt and Smith, 1998; Lavrov et al., 2002; Shi et al., 2014). The recombination model is only appropriate for the exchange and inversion of small fragments (Dowton and Campbell, 2001; Kong et al., 2009); thus, this recombination model was not suited to explaining the mitochondrial gene rearrangement of *Garra*. Regarding the two models, TDNL and DRRL, they are often used to explain mitochondrial gene rearrangements, but with genes aggregated in the same pole (encoded by the L- or H-strand) and with no change in relative order. Therefore, neither model can be used to explain the rearrangement mechanism that occurred in this study. The TDRL model was due to incomplete deletion of duplicated genes resulting in intergenic spacer regions or pseudogenes (Shi et al., 2015; Gong et al., 2019). This TDRL phenotype explains well the gene rearrangements with redundant genes. Evidence for the TDRL model was the presence of pseudogenes or duplicate genes and the location of gene spacer regions. Therefore, in the present study, the TDRL model was recommended to study rearrangement events, as gene rearrangements with duplicate CRs were observed in the mitochondrial genome of *Garra*.

Based on the putative model described above, we inferred the regions of mitochondrial gene rearrangement in three *Garra* species. First, normal mitochondrial gene blocks (*T-P-CR*) form gene blocks (*T-P-CR-T-P-CR*) after complete duplication (Figure 5A). Subsequently, after successive replications, some genes (*T* and *P*) undergo random loss events, leaving only an incompletely replicated CR (Figure 5B). Thus, after this replication and random loss, both CRs are preserved (Figure 5C).

### Phylogenetic Analysis

**FIGURE 6:**
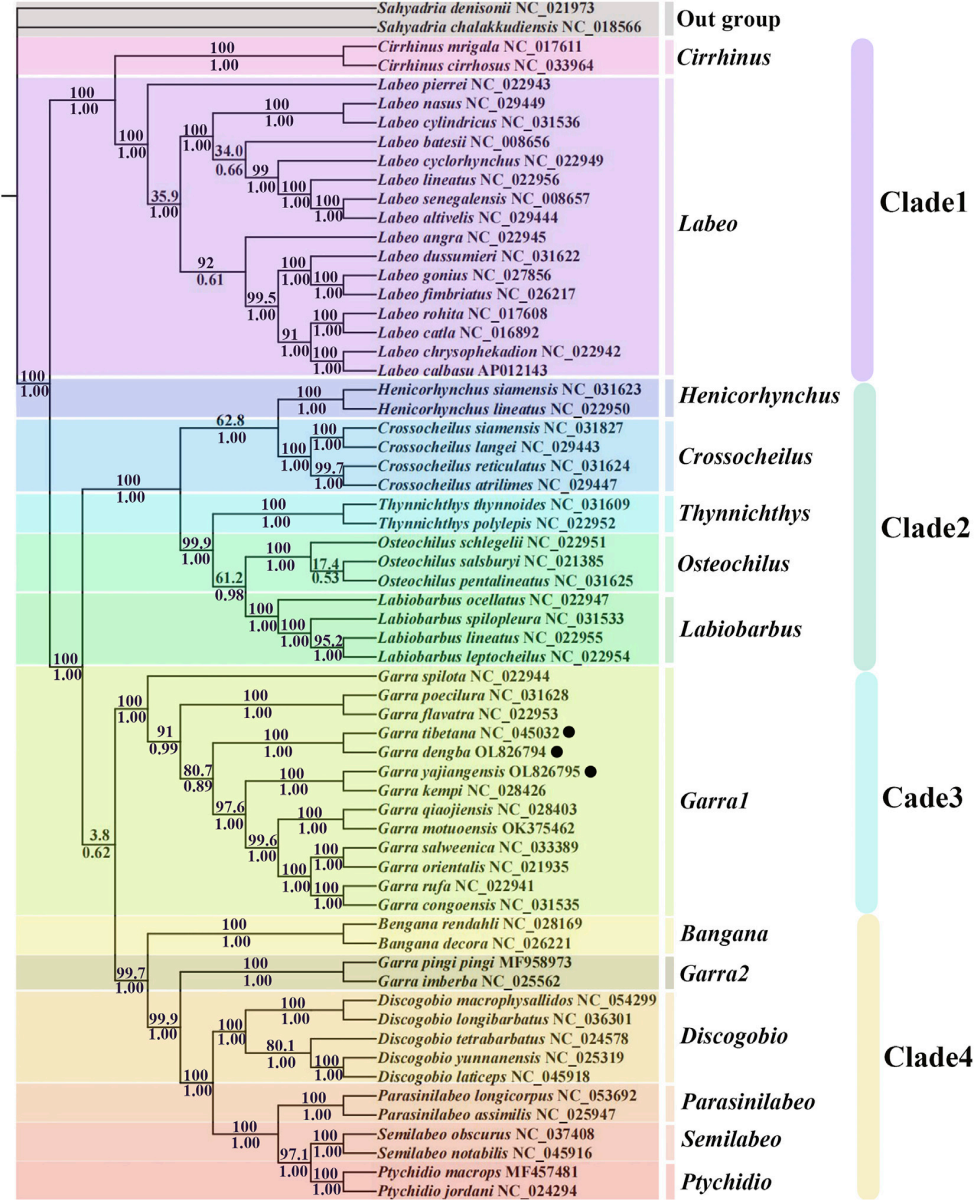
Phylogenetic analysis is based on the nucleotide sequences of the 12 PCGs in the mitogenome. The numbers beside the nodes are posterior probabilities (BI, bottom) and bootstrap (ML, top). Black point: gene rearrangement occurs.

To further study the evolutionary status of *G. dengba*, *G. tibetana,* and *G. yajiangensis* in the Labeoninae, we selected 58 closely related subfamilies and two outgroups (*Sahyadria chalakkudiensis* and *Sahyadria denisonii*) to construct evolutionary trees (BI and ML) to analyze phylogenetic relationships (Table 3). The results showed that the ML tree and the BI tree have the same topological structure. Thus, only one topology (BI) is displayed. In addition, the support values of BI tree are higher than ML tree (Figure 6). The phylogenetic trees showed that the subfamily Labeoninae is divided into three major clades and 13 genera (*Cirrhinus*, *Labeo*, *Henicorhynchus*, *Crossocheilus*, *Thynnichthys*, *Osteochilus*, *Labiobarbus*, *Garra*, *Bangana*, *Discogobio Parasinilabeo*, *Semilabeo*, *Ptychidio*). First, the clade1, genera *Cirrhinus,* and *Labeo* show a sister relationship, both of them were once classified in the genus *Labeonini* (Yang et al., 2012). Then, the clade2, including *Henicorhynchus*, *Crossocheilus*, *Thynnichthys*, *Osteochilus*, and *Labiobarbus*, which belonged to “*Osteochilini*” (Saenjundaeng et al., 2020). The clade3 aggregates all mitochondrial genes with rearrangements in the genus *Garra*. Except for *Bangana* and *Garra*2, all other species of the fourth clade used to belong to the genus *Semilabeonini* (Stout et al., 2016). The genus *Bangana* belonged to the cyprinid tribe *Labeonini* too (Zhang and Chen, 2006; Kottelat, 2017).

**TABLE 3:** List of 61 Labeoninae species and two outgroups used in this paper.

Organism	Family	Subfamily	Genus	Length	Accession NO.
*Bangana decora*	Cyprinidae	Labeoninae	*Bangana*	16607	NC_026221
*Bengana rendahli*	Cyprinidae	Labeoninae	*Bengana*	16586	NC_028169
*Cirrhinus cirrhosus*	Cyprinidae	Labeoninae	*Cirrhinus*	16588	NC_033964
*Cirrhinus mrigala*	Cyprinidae	Labeoninae	*Cirrhinus*	16600	NC_017611
*Crossocheilus atrilimes*	Cyprinidae	Labeoninae	*Crossocheilus*	16586	NC_029447
*Crossocheilus langei*	Cyprinidae	Labeoninae	*Crossocheilus*	16569	NC_029443
*Crossocheilus reticulatus*	Cyprinidae	Labeoninae	*Crossocheilus*	16588	NC_031624
*Crossocheilus siamensis*	Cyprinidae	Labeoninae	*Crossocheilus*	16611	NC_031827
*Discogobio laticeps*	Cyprinidae	Labeoninae	*Discogobio*	16596	NC_045918
*Discogobio longibarbatus*	Cyprinidae	Labeoninae	*Discogobio*	16594	NC_036301
*Discogobio macrophysallidos*	Cyprinidae	Labeoninae	*Discogobio*	16593	NC_054299
*Discogobio tetrabarbatus*	Cyprinidae	Labeoninae	*Discogobio*	16596	NC_024578
*Discogobio yunnanensis*	Cyprinidae	Labeoninae	*Discogobio*	16602	NC_025319
*Garra congoensis*	Cyprinidae	Labeoninae	*Garra*	16761	NC_031535
*Garra dengba*	Cyprinidae	Labeoninae	*Garra*	16876	OL826794
*Garra flavatra*	Cyprinidae	Labeoninae	*Garra*	16743	NC_022953
*Garra imberba*	Cyprinidae	Labeoninae	*Garra*	16600	NC_025562
*Garra kempi*	Cyprinidae	Labeoninae	*Garra*	17104	NC_028426
*Garra motuoensis*	Cyprinidae	Labeoninae	*Garra*	16806	OK375462
*Garra orientalis*	Cyprinidae	Labeoninae	*Garra*	17288	NC_021935
*Garra pingi pingi*	Cyprinidae	Labeoninae	*Garra*	16599	MF958973
*Garra poecilura*	Cyprinidae	Labeoninae	*Garra*	16978	NC_031628
*Garra qiaojiensis*	Cyprinidae	Labeoninae	*Garra*	17096	NC_028403
*Garra rufa*	Cyprinidae	Labeoninae	*Garra*	16763	NC_022941
*Garra salweenica*	Cyprinidae	Labeoninae	*Garra*	16960	NC_033389
*Garra spilota*	Cyprinidae	Labeoninae	*Garra*	16822	NC_022944
*Garra tibetana*	Cyprinidae	Labeoninae	*Garra*	16861	NC_045032
*Garra yajiangensis*	Cyprinidae	Labeoninae	*Garra*	16835	OL826795
*Henicorhynchus lineatus*	Cyprinidae	Labeoninae	*Henicorhynchus*	16825	NC_022950
*Henicorhynchus siamensis*	Cyprinidae	Labeoninae	*Henicorhynchus*	17010	NC_031623
*Labeo altivelis*	Cyprinidae	Labeoninae	*Labeo*	16603	NC_029444
*Labeo angra*	Cyprinidae	Labeoninae	*Labeo*	16606	NC_022945
*Labeo batesii*	Cyprinidae	Labeoninae	*Labeo*	16603	NC_008656
*Labeo calbasu*	Cyprinidae	Labeoninae	*Labeo*	16607	AP012143
*Labeo catla*	Cyprinidae	Labeoninae	*Labeo*	16590	NC_016892
*Labeo chrysophekadion*	Cyprinidae	Labeoninae	*Labeo*	16602	NC_022942
*Labeo cyclorhynchus*	Cyprinidae	Labeoninae	*Labeo*	16610	NC_022949
*Labeo cylindricus*	Cyprinidae	Labeoninae	*Labeo*	16602	NC_031536
*Labeo dussumieri*	Cyprinidae	Labeoninae	*Labeo*	16613	NC_031622
*Labeo fimbriatus*	Cyprinidae	Labeoninae	*Labeo*	16614	NC_026217
*Labeo gonius*	Cyprinidae	Labeoninae	*Labeo*	16614	NC_027856
*Labeo lineatus*	Cyprinidae	Labeoninae	*Labeo*	16606	NC_022956
*Labeo nasus*	Cyprinidae	Labeoninae	*Labeo*	16601	NC_029449
*Labeo pierrei*	Cyprinidae	Labeoninae	*Labeo*	16766	NC_022943
*Labeo rohita*	Cyprinidae	Labeoninae	*Labeo*	16626	NC_017608
*Labeo senegalensis*	Cyprinidae	Labeoninae	*Labeo*	16604	NC_008657
*Labiobarbus leptocheilus*	Cyprinidae	Labeoninae	*Labiobarbus*	16593	NC_022954
*Labiobarbus lineatus*	Cyprinidae	Labeoninae	*Labiobarbus*	16598	NC_022955
*Labiobarbus ocellatus*	Cyprinidae	Labeoninae	*Labiobarbus*	16596	NC_022947
*Labiobarbus spilopleura*	Cyprinidae	Labeoninae	*Labiobarbus*	16593	NC_031533
*Osteochilus pentalineatus*	Cyprinidae	Labeoninae	*Osteochilus*	16563	NC_031625
*Osteochilus salsburyi*	Cyprinidae	Labeoninae	*Osteochilus*	16599	NC_021385
*Osteochilus schlegelii*	Cyprinidae	Labeoninae	*Osteochilus*	16575	NC_022951
*Parasinilabeo assimilis*	Cyprinidae	Labeoninae	*Parasinilabeo*	16602	NC_025947
*Parasinilabeo longicorpus*	Cyprinidae	Labeoninae	*Parasinilabeo*	16596	NC_053692
*Ptychidio jordani*	Cyprinidae	Labeoninae	*Ptychidio*	16602	NC_024294
*Ptychidio macrops*	Cyprinidae	Labeoninae	*Ptychidio*	16604	MF457481
*Sahyadria chalakkudiensis*	Cyprinidae	Smiliogastrinae	*Sahyadria*	16989	NC_018566
*Sahyadria denisonii*	Cyprinidae	Smiliogastrinae	*Sahyadria*	16899	NC_021973
*Semilabeo notabilis*	Cyprinidae	Labeoninae	*Semilabeo*	16599	NC_045916
*Semilabeo obscurus*	Cyprinidae	Labeoninae	*Semilabeo*	16598	NC_037408
*Thynnichthys polylepis*	Cyprinidae	Labeoninae	*Thynnichthys*	16599	NC_022952
*Thynnichthys thynnoides*	Cyprinidae	Labeoninae	*Thynnichthys*	16589	NC_031609

In addition, four clades in this phylogenetic tree show high support values except for the value between clade3 and clade4 (posterior probabilities = 0.62; bootstrap = 3.8; Figure 6), indicating that these two clades in Labeoninae are not highly differentiated. All mitochondrial sequences of genus *Garra* in clade3 have two control regions (Figure 5C) (Li et al., 2016; Su et al., 2015; Xiong et al., 2016; Li et al., 2016), while *G. pingi pingi* and *G. imberba* have only one control region and clusters with other Labeoninae species in clade4 without rearrangements, which is consistent with previous studies (Figure 5A) (Zou et al., 2018). Furthermore, each of the 13 different *Garra* fishes in clade3 clustered into six pairs of sister branches, with only *G. spilota* forming a monophyletic branch. The three species from Tibet described in this study (species marked by dots in the tree), *G. tibetana* and *G. dengba*, form sister branches, showing the close relationship between them. *G. yajiangensis* and *G. kempi* cluster together, while *G. qiaojiensis* showed a close relationship with *G. kempi* in Zou et al. (2018)’ s study, which may be limited by the number of mitochondria in *Garra* (Zou et al., 2018). It is certain that *Garra*1 and *Garra*2 are distantly related due to the rearrangement of mitochondria in *Garra*1 species, whereas *Garra*2 species are not rearranged. Thus, we also found that the species of *Garra*2 are all from Sichuan (He et al., 2016; Zou et al., 2018), and we speculated that none mitochondria of *Garra* in this region have two control regions or other rearrangements. However, due to the lack of *Garra* samples from Sichuan, further sample collection is needed to confirm this conjecture.

## Conclusion

With the development of genetic investigations, the reliance on molecular info is gradually surpassing morphological data, and molecular assays have become the most used method in the study of biological system development. Therefore, in this study, we sequenced and assembled the complete mitochondrial gene of *G. dengba*, *G. tibetana,* and *G. yajiangensis* and determined its characteristics, which contains 37 genes and two control regions. Upon comparison with typical vertebrate mitochondrial genes, we discovered a clear rearrangement of mitochondrial genes in *G. dengba*, *G. tibetana*, and *G. yajiangensis*, the position of *tRNA-Pro* in the mitochondrial genome of these three *Garra* species changed, accompanied by CR repeat. The TDRL model is most suitable to explain the gene rearrangements with redundant genes in this study. The two phylogenetic trees (BI and ML) were generated based on the mitochondrial genomes of 61 Labeoninae. Both phylogenetic trees strongly favor the non-monophyly of *Garra*, which were divided into two different clades (rearrangement and no rearrangement), providing a more advanced classification of Labeoninae. Further, our findings provide a theoretical ground for a deeper understanding of the mechanism and evolution of genus *Garra* gene rearrangement and phylogenetic studies of Labeoninae.

## Data Availability

The raw data is publicly accessible at GenBank under Bioproject accession number PRJNA837076 and the annotation sequences were submitted to NCBI (G. dengba, accession no. OL826794; G. tibetana accession no. NC_045032 and G. yajiangensis, accession no. OL826795).
