# Supplementary figures and images for: Novel Gene Rearrangement in the Mitochondrial Genome of Three Garra and Insights Into the Phylogenetic Relationships of Labeoninae
Source: Front Genet. 2022 Jun 8;13:922634. doi: 10.3389/fgene.2022.922634 (PMC9213810; doi:10.3389/fgene.2022.922634)

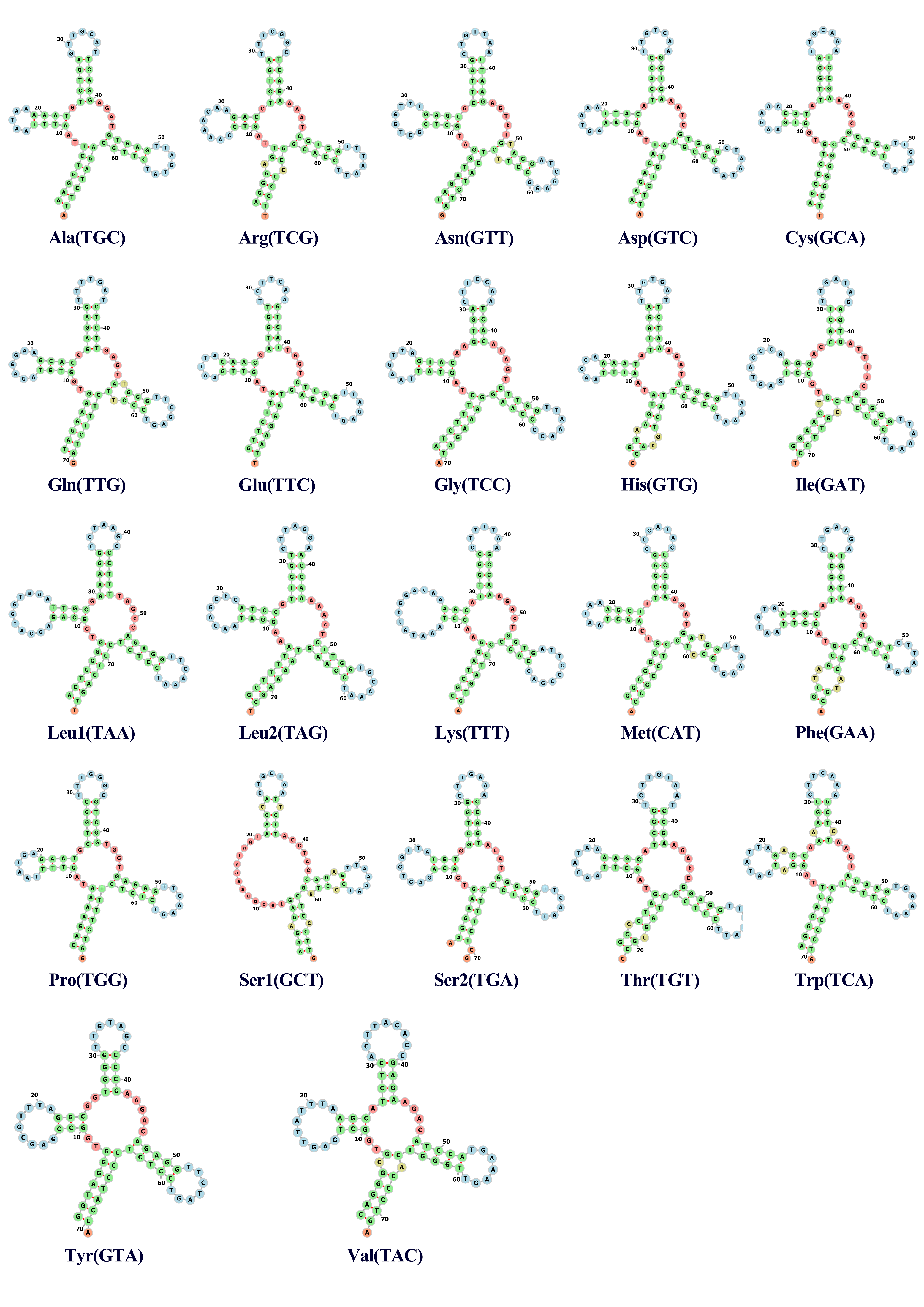

Supplement: Supplementary file 1 [file Image3.TIF]

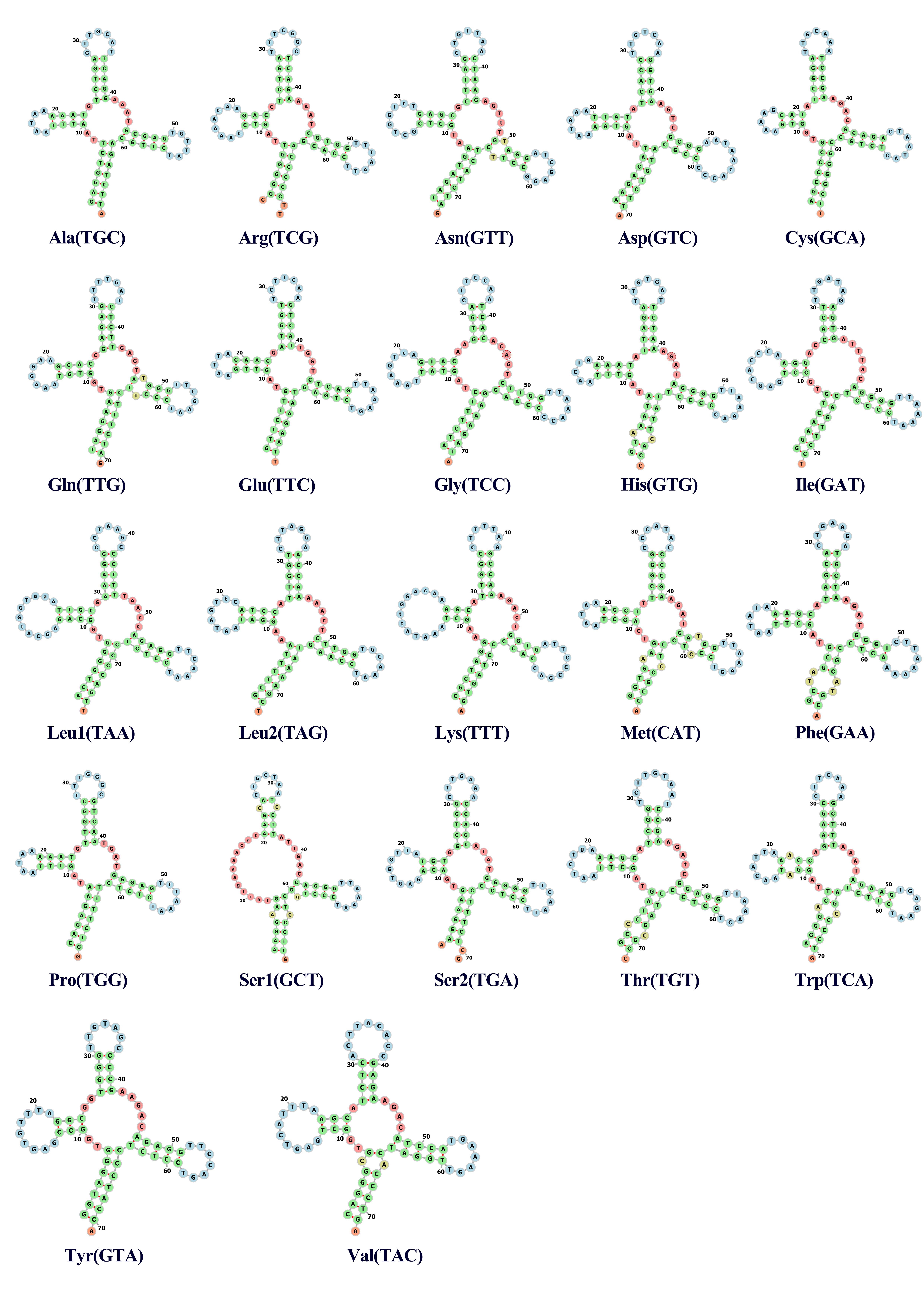

Supplement: Supplementary file 2 [file Image4.TIF]

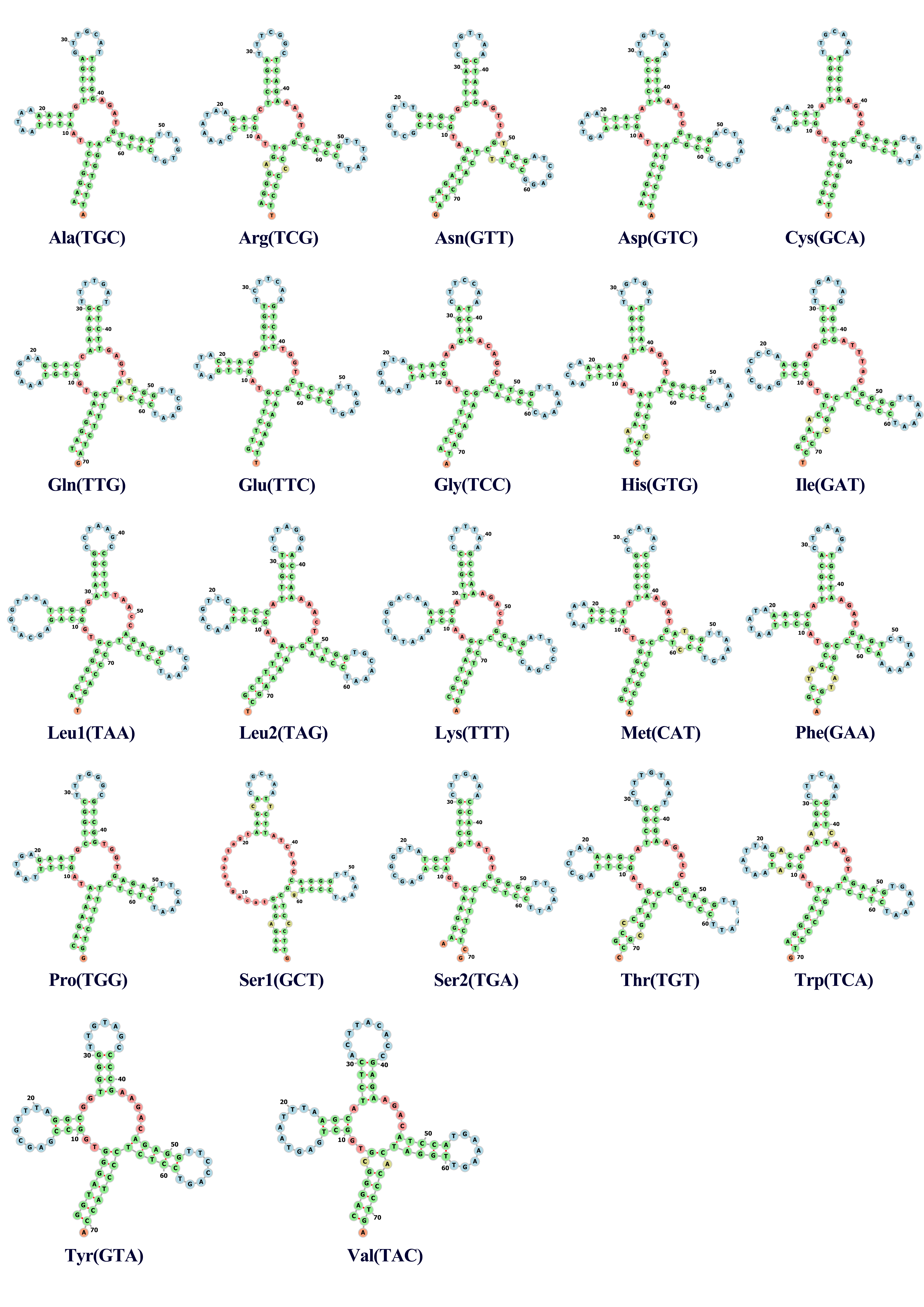

Supplement: Supplementary file 3 [file Image2.TIF]

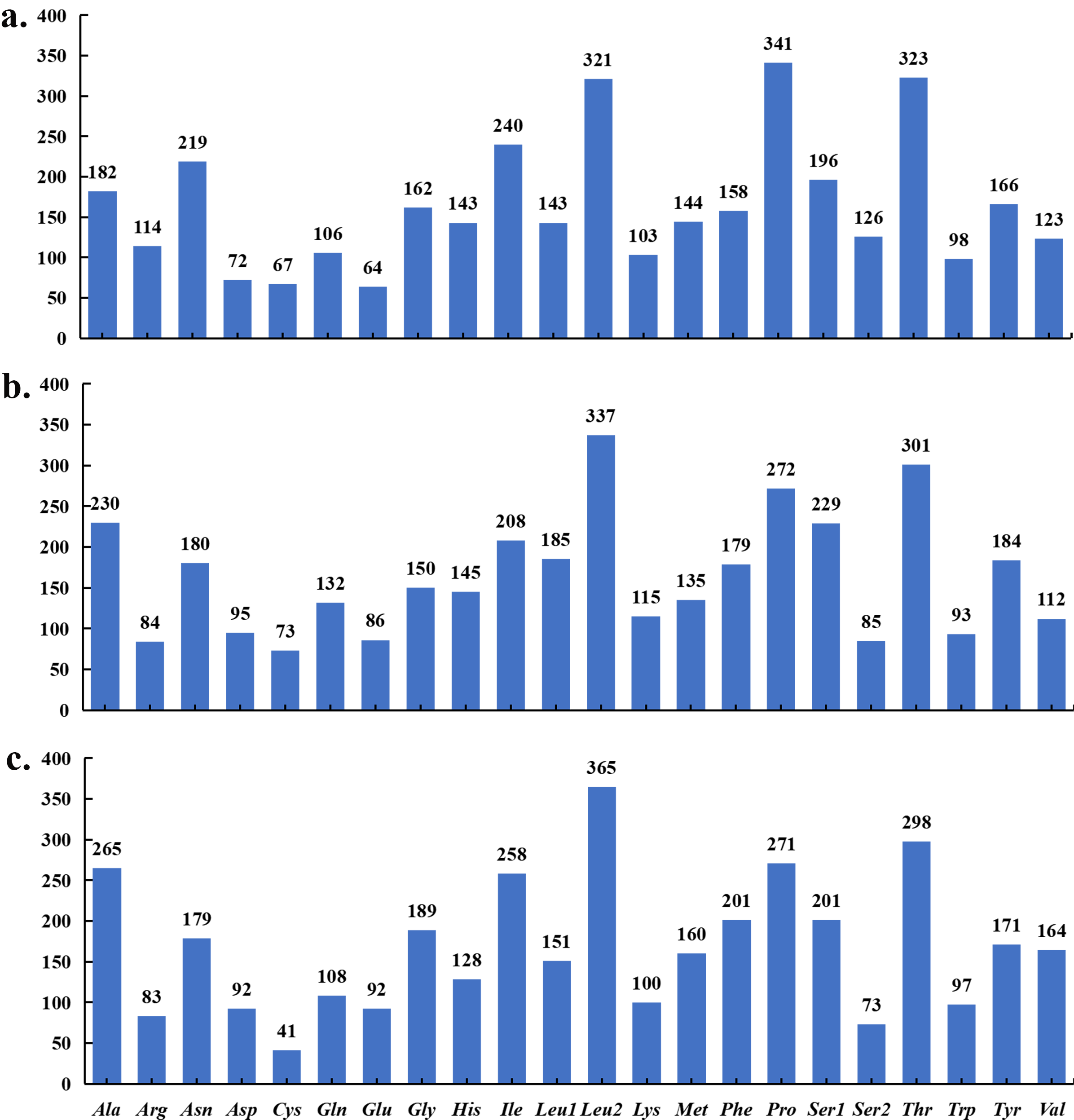

Supplement: Supplementary file 4 [file Image1.TIF]
