# Supplementary material for: Novel Gene Rearrangement in the Mitochondrial Genome of Three Garra and Insights Into the Phylogenetic Relationships of Labeoninae
Source: Front Genet. 2022 Jun 8;13:922634. doi: 10.3389/fgene.2022.922634 (PMC9213810; doi:10.3389/fgene.2022.922634)
Supplement: Supplementary file 5 [file Table5.DOCX]

Table S2. Composition and skewness of *Garra* *yajiangensis* mitogenome.

| **Gene** | **A** | **T** | **C** | **G** | **A+T(%)** | **G+C(%)** | **AT-skew** | **GC-skew** | **Length (bp)** |
| --- | --- | --- | --- | --- | --- | --- | --- | --- | --- |
| **Mitogenome** | 30.48 | 28.21 | 26.62 | 14.70 | 58.68 | 41.32 | 0.0387 | -0.2885 | 11,409 |
| **ND1** | 30.77 | 27.38 | 28.41 | 13.44 | 58.15 | 41.85 | 0.0582 | -0.3578 | 975 |
| **ND2** | 34.86 | 23.30 | 30.75 | 11.08 | 58.17 | 41.83 | 0.1987 | -0.4703 | 1,047 |
| **COI** | 27.98 | 30.63 | 24.50 | 16.89 | 58.61 | 41.39 | -0.0451 | -0.1838 | 1,551 |
| **COII** | 32.56 | 26.63 | 26.19 | 14.62 | 59.19 | 40.81 | 0.1002 | -0.2837 | 691 |
| **ATP8** | 35.76 | 27.27 | 26.06 | 10.91 | 63.03 | 36.97 | 0.1346 | -0.4098 | 165 |
| **ATP6** | 29.39 | 30.41 | 26.32 | 13.89 | 59.80 | 40.20 | -0.0171 | -0.3091 | 684 |
| **COIII** | 28.50 | 26.72 | 28.50 | 16.28 | 55.22 | 44.78 | 0.0323 | -0.2727 | 786 |
| **ND3** | 30.09 | 29.51 | 27.51 | 12.89 | 59.60 | 40.40 | 0.0096 | -0.3617 | 349 |
| **ND4L** | 27.61 | 25.25 | 32.32 | 14.81 | 52.86 | 47.14 | 0.0446 | -0.3714 | 297 |
| **ND4** | 32.15 | 27.37 | 27.01 | 13.47 | 59.52 | 40.48 | 0.0803 | -0.3345 | 1,381 |
| **ND5** | 34.16 | 26.64 | 27.30 | 11.90 | 60.80 | 39.20 | 0.1235 | -0.3930 | 1,824 |
| **ND6** | 12.26 | 41.38 | 12.07 | 34.29 | 53.64 | 46.36 | -0.5429 | 0.4793 | 522 |
| **Cytb** | 30.87 | 28.76 | 26.74 | 13.63 | 59.63 | 40.37 | 0.0354 | -0.3246 | 1,137 |
| **rRNAs** | 35.95 | 20.17 | 24.16 | 19.72 | 56.12 | 43.88 | 0.2811 | -0.1011 | 2,637 |
| **tRNAs** | 29.06 | 27.13 | 20.97 | 22.84 | 56.19 | 43.81 | 0.0342 | 0.0425 | 1,559 |
| **CR1** | 33.37 | 32.39 | 20.24 | 14.00 | 65.75 | 34.25 | 0.0150 | -0.1821 | 914 |
| **CR2** | 44.57 | 28.84 | 19.85 | 6.74 | 73.41 | 26.59 | 0.2143 | -0.4930 | 267 |
