# Supplementary material for: Novel Gene Rearrangement in the Mitochondrial Genome of Three Garra and Insights Into the Phylogenetic Relationships of Labeoninae
Source: Front Genet. 2022 Jun 8;13:922634. doi: 10.3389/fgene.2022.922634 (PMC9213810; doi:10.3389/fgene.2022.922634)
Supplement: Supplementary file 6 [file Table4.DOCX]

Table S1. Composition and skewness of *Garra tibetana* mitogenome.

| **Gene** | **A** | **T** | **C** | **G** | **A+T(%)** | **G+C(%)** | **AT-skew** | **GC-skew** | **Length (bp)** |
| --- | --- | --- | --- | --- | --- | --- | --- | --- | --- |
| **Mitogenome** | 32.31 | 26.13 | 15.35 | 26.20 | 58.44 | 41.56 | 0.1057 | -0.2610 | 16,861 |
| **ND1** | 30.67 | 26.05 | 13.95 | 29.33 | 56.72 | 43.28 | 0.0814 | -0.3555 | 975 |
| **ND2** | 34.07 | 24.02 | 11.96 | 29.95 | 58.09 | 41.91 | 0.1730 | -0.4292 | 1,045 |
| **COI** | 27.40 | 30.75 | 17.34 | 24.50 | 58.16 | 41.84 | -0.0576 | -0.1710 | 1,551 |
| **COII** | 31.40 | 27.50 | 15.77 | 25.33 | 58.90 | 41.10 | 0.0663 | -0.2324 | 691 |
| **ATP8** | 36.14 | 28.92 | 10.24 | 24.70 | 65.06 | 34.94 | 0.1111 | -0.4138 | 166 |
| **ATP6** | 30.07 | 30.22 | 13.28 | 26.42 | 60.29 | 39.71 | -0.0024 | -0.3309 | 685 |
| **COIII** | 28.66 | 26.11 | 16.18 | 29.04 | 54.78 | 45.22 | 0.0465 | -0.2845 | 785 |
| **ND3** | 28.29 | 29.71 | 14.86 | 27.14 | 58.00 | 42.00 | -0.0246 | -0.2925 | 350 |
| **ND4L** | 25.84 | 25.84 | 16.44 | 31.88 | 51.68 | 48.32 | 0.0000 | -0.3194 | 298 |
| **ND4** | 32.73 | 27.23 | 13.11 | 26.94 | 59.96 | 40.04 | 0.0918 | -0.3454 | 1,381 |
| **ND5** | 33.66 | 27.03 | 12.23 | 27.08 | 60.69 | 39.31 | 0.1093 | -0.3780 | 1,824 |
| **ND6** | 13.98 | 42.34 | 31.80 | 11.88 | 56.32 | 43.68 | -0.5034 | 0.4561 | 522 |
| **Cytb** | 29.36 | 29.27 | 14.55 | 26.82 | 58.63 | 41.37 | 0.0015 | -0.2966 | 1,141 |
| **rRNAs** | 28.49 | 27.47 | 23.41 | 20.62 | 55.96 | 44.04 | 0.0181 | 0.0634 | 1,576 |
| **tRNAs** | 35.24 | 20.50 | 20.31 | 23.95 | 55.74 | 44.26 | 0.2644 | -0.0822 | 2,639 |
| **CR1** | 34.07 | 32.30 | 20.53 | 13.10 | 66.37 | 33.63 | 0.0268 | -0.2211 | 901 |
| **CR2** | 41.86 | 29.57 | 22.59 | 5.98 | 71.43 | 28.57 | 0.1721 | -0.5814 | 301 |
